# Treatment approaches to horses with acute diarrhea admitted to referral institutions: A multicenter retrospective study

**DOI:** 10.1371/journal.pone.0313783

**Published:** 2024-11-20

**Authors:** Diego E. Gomez, Jamie J. Kopper, David P. Byrne, David L. Renaud, Angelika Schoster, Bettina Dunkel, Luis G. Arroyo, Anna Mykkanen, William F. Gilsenan, Tina H. Pihl, Gabriela Lopez-Navarro, Brett S. Tennent-Brown, Laura D. Hostnik, Mariano Mora-Pereira, Fernando Marques, Jenifer R. Gold, Sally L. DeNotta, Isabelle Desjardins, Allison J. Stewart, Taisuke Kuroda, Emily Schaefer, Olimpo J. Oliver-Espinosa, Gustavo Ferlini Agne, Benjamin Uberti, Pablo Veiras, Katie M. Delph Miller, Rodolfo Gialleti, Emily John, Ramiro E. Toribio

**Affiliations:** 1 Department of Clinical Studies, Ontario Veterinary College, University of Guelph, Guelph, ON, Canada; 2 Department of Veterinary Clinical Sciences, Iowa State University College of Veterinary Medicine, Ames, Iowa, United States of America; 3 School of Veterinary Medicine, Murdoch University, Perth, WA, Australia; 4 Department of Population Medicine, Ontario Veterinary College, University of Guelph, Guelph, ON, Canada; 5 Vetsuisse Faculty, Equine Department University of Zurich, Zurich, Switzerland; 6 Ludwig-Maximilians-University Munich, Equine Clinic, Oberschleissheim, Germany; 7 Department of Clinical Science and Services, The Royal Veterinary College, Hertfordshire, United Kingdom; 8 Department of Veterinary Biosciences, Faculty of Veterinary Medicine, University of Helsinki, Helsinki, Finland; 9 Rood and Riddle Equine Hospital, Lexington, KY, United States of America; 10 Department of Veterinary Clinical Sciences, Faculty of Health and Medical Sciences, University of Copenhagen, Taastrup, Denmark; 11 Departamento de Medicina, Cirugía y Zootecnia Equina, Facultad de Medicina Veterinaria y de Zootecnia, Universidad Nacional Autonoma de Mexico, Ciudad de Mexico, Mexico; 12 U-Vet Werribee Animal Hospital and Faculty of Veterinary and Agricultural Sciences, The University of Melbourne, Werribee, Victoria, Australia; 13 College of Veterinary Medicine, The Ohio State University, Columbus, Ohio, United States of America; 14 Department of Clinical Sciences, College of Veterinary Medicine, Auburn University, Auburn, AL, United States of America; 15 Department of Medical Sciences, School of Veterinary Medicine, University of Wisconsin-Madison, Madison, WI, United States of America; 16 Department of Veterinary Clinical Sciences, Washington State University, Pullman, WA, United States of America; 17 Wisconsin Equine Clinic and Hospital, Oconomowoc, WI, United States of America; 18 Department of Large Animal Clinical Sciences, College of Veterinary Medicine, University of Florida, Gainesville, FL, United States of America; 19 University of Lyon, VetAgro Sup, GREMERES-ICE Lyon Equine Research Center, Marcy l’Etoile, France; 20 School of Veterinary Science, The University of Queensland, Gatton, Queensland, Australia; 21 Clinical Veterinary Medicine Division, Equine Research Institute, Japan Racing Association, Shimotsuke, Tochigi, Japan; 22 Department of Large Animal Clinical Sciences, Marion duPont Scott Equine Medical Center, Leesburg, VA, United States of America; 23 Clinica de Grandes Animales, Departamento de Salud Animal, Facultad de Medicina Veterinaria y de Zootecnia, Universidad Nacional de Bogota de Santa Fe, Bogota, Colombia; 24 School of Animal and Veterinary Science, Roseworthy Campus, The University of Adelaide, South Australia, Australia; 25 Instituto de Ciencias Clinicas Veterinarias, Facultad de Ciencias Veterinarias, Universidad Austral de Chile, Valdivia, Chile; 26 Equine Veterinary Medical Center, Qatar Foundation, Doha, Qatar; 27 Fethard Equine Hospital, Fethard, Tipperary, Ireland; 28 Department of Clinical Sciences, Kansas State University, College of Veterinary Medicine, Manhattan, KS, United States of America; 29 Centro di Ricerca del Cavallo Sportivo, Department of Veterinary Medicine, University of Perugia, Perugia, Italy; 30 Department of Health Management, Atlantic Veterinary College, University of Prince Edward Island, Charlottetown, PE, United States of America; Michigan State University, UNITED STATES OF AMERICA

## Abstract

**Background:**

This study aimed to describe and compare therapeutic approaches for horses with acute diarrhea in different geographic regions worldwide.

**Methods:**

Clinical information was retrospectively collected from diarrheic horses presented to participating institutions between 2016 and 2020, including fluid therapy on admission, antimicrobial drugs, probiotics, anti-endotoxic medications, anti-inflammatory drugs, gastroprotectants, digital cryotherapy, and toxin-binding agents. Seasonal and geographic differences were investigated.

**Results:**

1438 horses from 26 participating hospitals from 5 continents were included. On admission, 65% (926/1419) of horses were administered a fluid bolus. Antimicrobial drugs were administered to 55% (792/1419) within the first 24 hours of admission, with penicillin and gentamicin being the most used combination (25%, 198/792). Horses with leukopenia (OR: 2.264, 95%CI: 1.754 to 2.921; P<0.001) or meeting systemic inflammatory response syndrome criteria (OR: 2.542, 95%CI: 1.919 to 3.368; P<0.001) had higher odds of being administered antimicrobial drugs. Other treatments administered included probiotics (15%, 215/1438), polymyxin B (13%; 187/1438), pentoxifylline (8%; 118/1438), gastroprotectants (44%; 626/1419), digital cryotherapy (34%; 489/1435), plasma transfusion (13%; 182/1410) and toxin-binding agents (36%; 515/1438).

**Limitations:**

Due to the retrospective nature of the study, the rationale for treatment decisions was unavailable, and associations with survival could not be evaluated.

**Conclusions:**

Treatments varied between hospitals from different geographic regions. Prospective clinical trials are required to evaluate the effects of various treatments on survival.

## Introduction

Acute diarrhea is a common life-threatening cause of hospitalization in horses [1–3]. It is associated with significant morbidity due to complications, including sepsis, laminitis, jugular vein thrombophlebitis, and acute renal injury [1, 3–5]. Many complications related to diarrhea in the horse are due to high fluid and electrolyte losses and disruption of the intestinal mucosal barrier, resulting in the translocation of intraluminal bacteria and their by-products, leading to systemic inflammation or sepsis [1, 6–8]. Thus, treatments that effectively and rapidly improve the hemodynamic status, restore intestinal mucosal barrier function, and prevent or treat systemic inflammation and sepsis are recommended [9]. However, few prospective clinical trials have evaluated the effect of the different therapies on the outcome of horses with diarrhea. Many treatments currently employed by clinicians for managing diarrheic horses are based on anecdotal opinions, extrapolated from studies in humans or small-scale retrospective or prospective studies, often with conflicting results. Identifying the current treatments for diarrheic horses can have important implications in establishing guidelines for clinical practice and formulating research questions. Thus, this retrospective study aimed to describe treatment approaches used internationally for managing horses hospitalized in tertiary referral hospitals for acute diarrheal disease.

## Materials and methods

### Animals

This was a multicenter retrospective case series study. A convenience sample of university teaching hospitals and large private hospitals worldwide representative of the different geographic areas was contacted via email. The minimum number of horses required to participate was 30 cases admitted to each institution between 2016 and 2020. Inclusion criteria comprised horses > 1 year old presenting for acute diarrhea < 48h of onset. Horses that developed diarrhea within the first 24 hours after admission were also included, but horses that underwent surgery and developed diarrhea post-operatively were excluded.

### Retrospective data collection

From each hospital record, demographic data (sex, breed, age), month, season (only for institutions from the Southern and Northern hemispheres (23.5° to 66.5° North and South of Equator, 0°) and year of presentation were collected. In the Northern Hemisphere, seasons were classified as winter (December, January, and February), spring (March, April, and May), summer (June, July, and August) and fall (September, October, and November). For the Southern hemisphere, seasons were classified as winter (June, July, and August), spring (September, October, and November), summer (December, January, and February) and fall (March, April, and May). Institutions were grouped into geographic areas (e.g., North America, Latin America, Europe, Australia, and Japan). Development of laminitis (yes or no) during hospitalization and survival to hospital discharge (yes or no) were also recorded. Records were reviewed between August 27, 2021, and March 1, 2022. Demographic information was retrieved from all records, but information that could identify individual participants during or after data collection was not collected. Data on physical examination findings, complete blood cell count (packed cell volume (PCV, %), total white blood cell count (WBC, cells/μL), neutrophil count (cells/μL), biochemistry profile (total calcium, tCa, mmol/L), ionized calcium (iCa, mmol/L), total protein (TP, g/dL), L-lactate (mmol/L), and creatinine concentrations (mg/dL) were collected. The causes of diarrhea were also retrospectively collected and presented elsewhere [10, 11]. The hydration status of the horses, subjectively determined by the attending clinician, was recorded as normal, mild, moderate, or severe dehydration. The presence or absence of systemic inflammatory response syndrome (SIRS) on presentation was determined based on previously published SIRS criteria in horses [12], where horses with SIRS are defined by those who met two or more of the following criteria: HR > 40 bpm, RR > 20 rpm, T > 38.5°C or < 36.5°C, and white blood cell (WBC) count < 5,300 or > 14,800 cells/μL. Data recorded regarding treatments employed included fluid therapy at admission (administration of hypertonic saline solution [HSS] (yes/no) or a bolus of crystalloids (type and volume)); administration of synthetic and natural colloids (type, duration, volume); administration of antimicrobial drugs at any point of hospitalization (type and length); administration of pentoxifylline (yes/no), polymyxin B (yes/no), probiotics (yes/no and type) and gastroprotectans (yes/no; type and mechanism of action (e.g., proton pump inhibitor [PPI] or H_2_ antagonist [H_2_])); administration of toxin binding agents (yes/no and type) and antidiarrheal drugs (yes/no and type), and application of cryotherapy for prevention of laminitis (yes/no and technique). Data regarding administration of non-steroidal anti-inflammatory drugs (NSAIDs) was not collected.

### Statistical analysis

The normality of the data was assessed using normal probability Q-Q plots and the Kolmogorov-Smirnov test, and data were analyzed accordingly. Descriptive statistics included mean, standard deviation (SD), median, and ranges.

Categorical variables (e.g., use of therapies in horses with specific health alterations) were compared between groups using *X*^2^ or Fisher’s exact tests. In contrast, continuous variables were compared with a Student’s *t*-test or the Wilcoxon test. In addition, categorical variables were compared among geographic areas (i.e., North America, Latin America, Europe, Australia, Japan) using the *X*^2^ or Fisher’s exact tests, and continuous variables were compared using a One-way ANOVA with the post-hoc Tukey Honestly Significant Difference test or the non-parametric Steel-Dwass test for multiple comparisons. Antimicrobial use rates were compared among geographic regions. *Neorickettsia risticii* is a pathogen present in North America and requires specific antimicrobial therapy with oxytetracycline. Therefore, antimicrobial use rates were also compared, excluding North America. Antimicrobial treatment rates were also compared between leukopenic and non-leukopenic horses that met or did not meet the SIRS criteria and horses that met the SIRS criteria with and without leukopenia. Odds ratios were calculated using multivariable mixed models. Sex and age were included as fixed effects and institution as random effects. A mixed multivariable model was used to assess the association between the administration of crystalloid fluid boluses on admission and the degree of dehydration, creatinine, total plasma and L-lactate concentration and PCV values. Sex and age were included as fixed effects and institution as random effects. A P-value < 0.05 was considered significant. Statistical analyses and figures were performed using statistical software (StataCorp. 2021. Stata Statistical Software: Release 17. College Station, TX: StataCorp LLC) and JMP (JMP 16, SAS Institute Inc., Cary, NC).

## Results

### Participating institutions

Among the invited institutions 40 agreed to participate, and 16 did not respond. After an initial review of their medical records, ten institutions concluded they did not have enough cases to contribute, and three indicated that they could not collect the data due to time constraints. In addition, one institution submitted a small number of cases with limited information for analysis and was excluded from the study. This left 26 institutions located in 14 different countries (Australia (n = 4), Canada (n = 2), Chile (n = 1), Colombia (n = 1), Denmark (n = 1), England (n = 1), France (n = 1), Ireland (n = 1), Italy (n = 1), Japan (n = 1), Mexico (n = 1), Norway (n = 1), Switzerland (n = 1), and the USA (n = 9) from 5 different geographic areas (North America, Latin America, Australia, Japan, and Europe) with cases presented between January 1, 2016, and December 31, 2020, for analysis. Institutions from North America included Auburn University (AU), University of Prince Edward’s Island (UPEI), Iowa State University (ISU), Kansas State University (KSU), Marion duPont Equine Medical Center (MdP), Rood and Riddle Equine Hospital (RREH), The Ohio State University (The OSU), University of Florida (UF), University of Guelph (UG), University of Wisconsin-Madison (UW), and Washington State University (WSU). Institutions from Europe included FETHARD equine hospital (FETHARD, Ireland), University of Copenhagen (Copenhagen), University of Helsinki (Helsinki), University of Lyon (Lyon), University of Perugia (Perugia), The Royal Veterinary College (RVC), and University of Zurich (Zurich). Australian institutions were The University of Adelaide (Adelaide), University of Melbourne (Melbourne), Murdoch University (Murdoch), and University of Queensland (UQ). Institutions from Latin America included Universidad Austral de Chile (AUCh), Universidad Nacional de Colombia (UNAL) and Universidad Nacional Autonoma de Mexico (UNAM). The Japan Racing Association Ritto Training Center (JRA Ritto) was included from Asia.

### Horses

A total of 1438 horses met the inclusion criteria. Of 1438, 630 (44%) were presented to institutions in North America, 483 (33%) in Europe, 149 (10%) in Latin America, 141 (10%) in Australia and 35 (2%) in Japan. The number and proportions of horses admitted in each institution and detailed information on presenting complaints, time of the year and other epidemiological information are reported elsewhere [10]. This study included 635 (47%) female and 763 (53%) male horses. The age of the horses ranged between 1 and 35 years (median 9 years). Thirty-four breeds were represented, with Thoroughbred (283/1438, 20%), Quarter Horses (203/1438, 17%), ponies (140/1438, 10%) and Draft horses (113/1438, 8%] being the most prevalent breeds.

### Complete blood count (CBC) and SIRS score

The complete description of the clinicopathological findings in the horses included in this study is presented elsewhere [10]. A total of 539/1413 horses [38%] had leukopenia (WBC < 5,300 cells/μL), and 60 [4.2%] had leukocytosis (WBC > 14,800 cells/μL). Information to calculate the SIRS score was available for 1118/1438 (78%) horses, with 800/1118 (66%) meeting the criteria for SIRS.

### Survival to hospital discharge

The overall survival proportion for diarrheic horses admitted to 26 institutions was 76% (1093/1438; 95%CI: 74% to 78%).

### Fluid therapy on admission

#### Hypertonic solution

Information regarding HSS administration was available for 1422 horses, with 15% (211/1422; 95%CI: 13% to 17%) of the horses receiving HSS on admission. The median administered volume of HSS was 2000 ml (range: 1000 to 7000). HSS was administered to only 1% (1/95) of the horses judged to be euhydrated, 5% (28/512) with mild, 25% (90/354) with moderate, and 50% (81/158) with severe dehydration (P < 0.05 for all comparisons). None of the horses in Japan received HSS. No differences were detected in the proportion of horses treated with HSS in Australia (18%), Europe (15%), North America (15%) and Latin America (13%) (P = 0.54).

#### Crystalloid fluid bolus

Information regarding the administration of crystalloid fluid bolus on admission was available for 1419 horses, with 65% (926/1419; 95%CI: 62% to 68%) of horses being administered a bolus. The median administered volume of crystalloids was 10 litres (0.5 to 90 litres). Crystalloid fluid boluses were administered to 38% (36/95) of euhydrated horses, 60% (306/512) of mildly dehydrated, 83% (294/354) of moderately dehydrated and 91% (142/158) of severely dehydrated (P < 0.05 for all comparisons).

The proportion of horses administered a fluid bolus in the different geographic areas is presented in **Table 1**. The type of crystalloids bolus administered was available for 891/1410 horses. Lactated Ringer’s solution (LRS) was used in 77% (687/891) of the cases, Plasmalyte 148 in 11% (100/891), Ringer’s acetate in 9% (81/891), 0.9% NaCl in 1% (12/891), and other solutions or combination of solutions in 1% (11/891). Horses with mild, moderate and severe dehydration had higher odds of receiving crystalloid fluid bolus on admission than those without dehydration. Also, increases in PCV values and creatinine concentrations were associated with higher odds of receiving crystalloid fluid bolus on admission (**Table 2**).

**Table 1:** The numbers and proportion of horses administered selected treatments during hospitalization to 1438 horses with diarrhea presented to institutions from North America, Latin America, Europe, Australia, and Japan.

Therapy	North America	Europe	Australia	Japan	Latin America
Crystalloid bolus on admission	61% [377/617]^a^	67% [322/481]^b^	65% [92/141]^ab^	68% [24/35]^ab^	76% [111/146]^c^
Synthetic Colloids	6% [41/613]^a^	17% [85/481]^b^	8% [12/138]^a^	23% [8/35]^b^	0.6% [1/144]^a^
Plasma Transfusion	15% [92/611]^a^	12% [160/481]^a^	8% [20/138]^a^	6% [2/35]^a^	5% [8/145]^b^
Antimicrobial therapy	61% [384/628]^a^	44% [210/469]^b^	52% [73/140]^b^	74% [26/35]^a^	66% [99/147]^a^
Polymyxin B	15% [93/630]^a^	9% [48/483]^a^	10% [5/141]^a^	86% [30/35]^b^	0.07% [1/149]^c^
Gastroprotectants	40% [251/619] ^c^	47% [226/480] ^a^	33% [47/139] ^c^	0% [0/35]	70% [102/146] ^b^
DTO Smectite	30% [188/625]^b^	36% [169/463]^b^	67% [94/141]^a^	35% [16/35]^b^	13% [19/144]^b^
Digital cryotherapy	45% [277/617]^a^	19% [89/481]^b^	73% [101/139]^c^	0% [0/35]	15% [22/145]^b^

Different letters within a row indicated a statistically significant difference (*P* < .05).

**Table 2:** Multivariable mixed model evaluating the association between the degree of dehydration, creatinine concentration and packet cell volume (PCV) and the administration of crystalloid fluid bolus at admission of horses with acute diarrhea.

		95% CI				95% CI	
	Estimate	Lower	Upper	p-value	OR	Lower	Upper
*Random effects*							
Institution	1.193	0.564	2.524	0.009	-	-	-
*Fixed effects*							
Dehydration							
None	Referent						
Mild	0.929	0.260	1.599	0.007	2.533	1.297	4.947
Moderate	1.857	1.079	2.635	<0.001	6.405	2.941	13.950
Severe	2.474	1.411	3.537	<0.001	11.669	4.100	34.359
PCV (%)	0.041	0.022	0.065	<0.001	1.042	1.017	1.067
Creatinine (mg/dL)	0.272	0.025	0.519	0.031	1.313	1.025	1.681

Note: Age and sex were included as fixed effects in all 3 models, but the effect was not significant (p > 0.05)

#### Synthetic colloids administration

Information on the administration of synthetic colloids within the first 24 hours after admission was available for 1410 horses, with 10% (147/1410; 95%CI: 9% to 12%) of horses receiving synthetic colloids. The median administered volume of synthetic colloids was 2 litres (range: 0.5 to 10 litres). Synthetic colloids were administered to 6% (6/95) of the horses judged as euhydrated, 6% (32/508) mildly dehydrated, 19% (67/353) moderately dehydrated, and 21% (33/154) severely dehydrated horses. The proportion of moderately and severely dehydrated horses receiving synthetic colloids was significantly higher than horses with mild or without dehydration (P < 0.01 for all comparisons). On admission, the median total solids/total plasma protein (TS/TPP) concentration was lower in horses administered synthetic colloids (50 g/L, IQR_25-75_: 44 to 60 g/L) than in horses not treated with colloids (62 g/L, IQR_25-75_: 56 to 70 g/L). The proportion of horses administered synthetic colloids in the different geographic areas is presented in **Table 1**. The type of colloids administered was available for 145/147 horses. Hydroxyethyl starch and succinylated gelatin solution were administered to 99% (142/144) and 1% (2/144), respectively.

#### Plasma transfusion during hospitalization

Information regarding administering plasma at any time during hospitalization was available for 1420 horses, with 13% (182/1410; 95%CI: 11% to 15%) of the horses having a transfusion. Overall, the median volume of plasma administered was 4 litres [0.5 to 18 litres]. However, due to inconsistencies with reported weights, likely due to inconsistent access to a scale in isolation facilities, a mL/kg dose could not be reported. The type of plasma administered was commercial plasma to 47% (86/182) of the horses, hospital-harvested plasma to 25% (45/182), and an unknown type of plasma to 28% (51/182).

The proportion of horses having a plasma transfusion during hospitalization was lower in Latin America than in the other geographic areas (**Tables 1 and 3)**. The median volume of plasma (commercial, harvested, or unknown) administered in Japan was 5.5 litres (range: 4.5 to 6.5 litres), Europe 5 litres (range: 1 to 18 litres), Latin America 3 litres (range: 2.5 to 3.5 litres), North America 3 litres (range: 0.5 to 17 litres), and Australia 2 litres (range: 1 to 18 litres). The median volume of plasma administered was higher in Europe than in Latin America, North America, and Australia (P < 0.05, for all comparisons). There were no statistical differences in the volume of harvested (IQ_25-75_: 3 to 6 litres) and commercial (IQ_25-75_: 2 to 5 litres) plasma administered to the horses (P = 0.143)

**Table 3:** Selected treatments administered during hospitalization to 1438 horses with diarrhea presented to 26 institutions from North America, Latin America, Europe, Australia, and Japan.

Institution [n]	Polymyxin B	Pentoxifylline	Gastroprotectants			DTO Smectite
			PPI	H_2_	Sucralfate	
Adelaide [n = 24]	25% [6/24]	8% [2/24]	42% [10/24]	-	-	92% [22/24]
Auburn [n = 47]	27% [12/45]	2% [1/45]	38% [17/45]	-	-	16% [7/45]
UPEI [n = 12]	-	-	42% [5/12]	-	-	-
UACh [n = 24]	-	-	42% [6/24]	-	-	-
Copenhagen [n = 110]	-	2% [2/108]	70% [77/110]*	55% [60/110]*	-	83% [91/110]
Fethard [n = 22]	18% [4/22]	-	55% [12/22]	-	-	18% [4/22]
Helsinki [n = 156]	0.6% [1/156]	-	4% [6/156]	44% [68/156]	-	26% [41/156]
Iowa [n = 30]	31% [9/29]	4% [1/29]	23% [7/30]	-		17% [5/30]
JRA/Ritto [n = 35]	86% [30/35]	-	-	-	-	46% [16/35]
KSU [n = 21]	30% [6/20]	20% [4/20]	45% [9/20]	-		35% [7/20]
Lyon [n = 37]	-	-	19% [7/37] ^#^	-	24% [9/37] ^#^	
MdP [n = 32]	28% [9/32]	9% [3/32]	63% [20/32]	-	-	59% [19/32]
Melbourne [n = 61]	-	-	8% [5/61]	2% [1/61]	13% [8/61]	74% [45/61]
Murdoch [n = 20]	-	10% [2/20]	65% [13/20]	-	-	70% [14/20]
The OSU [n = 56]	12% [7/54]	-	21% [12/56]	-	5% [3/56]	66% [37/56]
Perugia [n = 15]	-	-	7% [1/15]	-	13% [2/15]	-
RREH [n = 117]	29% [34/117]	36% [42/117]	69% [81/117]	-	-	48% [56/117]
RVC [n = 40]	13% [5/40]	-	10% [4/40]^^^	-	5% [2/40]^^^	50% [20/40]
UF [n = 38]	5% [2/37]	22% [8/37]	14% [5/37]	-	-	24% [9/37]
UG [n = 191]	0.5% [1/190]	2% [3/190]	0% [23/190]	0.5% [1/190]	21% [39/190]	2% [4/190]
UNAL [n = 31]	3% [1/31]	-	3% [1/31]	39% [12/31]	-	-
UNAM [n = 94]	-	45% [42/93]	40% [38/94]	48% [45/94]	-	20% [19/94]
UQ [n = 36]	25% [9/36]	-	11% [4/36]	-	-	36% [13/36]
UW [n = 44]	-	5% [2/41]	55% [24/44]	-	-	32% [14/44]
WSU [n = 42]	31% [13/42]	14% [6/42]	36% [15/42]	-	-	71% [30/42]
Zurich [n = 103]	37% [38/103]	-	29% [30/103]^‡^	-	17% [17/103]^‡^	[13/103]

Adelaide, The University of Adelaide; AU, Auburn University, UPEI, University of Prince Edward’s Island, AUCh, Universidad Austral de Chile; Copenhagen, University of Copenhagen; Fethard, Fethard equine hospital; Helsinki, University of Helsinki; ISU, Iowa State University; JRA Ritto, Japan Racing Association Ritto Training Center; KSU, Kansas State University; Lyon, University of Lyon; MdP, Marion duPont Scott Equine Medical Center; Melbourne, University of Melbourne; Murdoch, Murdoch University; The OSU, The Ohio State University; Perugia, University of Perugia; RREH, Rood and Riddle Equine Hospital; RVC, The Royal Veterinary College; UF, University of Florida; UG, University of Guelph; UNAL, Universidad Nacional de Colombia; UNAM, Universidad Nacional Autonoma de Mexico; UQ, University of Queensland; UW, University of Wisconsin-Madison; WSU, Washington State University; Zurich, University of Zurich. DTO, di-tri-octahedral; PPI, proton pump inhibitors; H2, Histamine H2 receptor antagonist. *53 horses were administered concurrently H2 and PPI

^#^6 horses were administered concurrently sucralfate and PPI

^^^2 horses were administered concurrently sucralfate and PPI

^‡^14 horses were administered concurrently sucralfate and PPI.

#### Antimicrobial treatment rates, regimens, and seasonality

Information regarding antimicrobial therapy was available for 1419 horses, with 55% (792/1419; 95%CI: 53% to 58%) of the horses being administered one or a combination of antimicrobial drugs within the first 24 hours of admission. The proportion of horses treated with antimicrobial drugs differed among institutions, varying from 17% to 94%. The proportion of horses treated with antimicrobial drugs was lower in Europe and Australia than in the other geographic areas (**Table 1)**. The overall median duration of antimicrobial therapy was 5 days (25 and 75% interquartile range: 3 to 7 days). The course of antimicrobial treatment was more prolonged in Australia (6 days, IQ_25-75_: 4 to 7 days) and Latin America (5.5 days, IQ_25-75_: 3 to 8 days) than in North America (4 days, IQ_25-75_: 3 to 6 days) and Europe (5 days, IQ_25-75_: 3 to 7 days) (P < 0.05).

The combinations of penicillin and gentamicin (25%, 198/792), penicillin, gentamicin, and metronidazole (15%, 121/792), and monotherapy with oxytetracycline (16%, 126/792) or metronidazole (12%, 94/792) accounted for 68% of all treatment regimens. Each of the remaining types of antimicrobial therapies accounted for less than 4%. **Table 4** lists the most common antimicrobial regimens used in the different geographic areas.

**Table 4:** Antimicrobial treatment regimens administered to 1438 horses with acute diarrhea presented to 26 institutions from North America, Latin America, Europe, Australia, and Japan.

Geographic region	Antimicrobial regimen	n	%
North America (n = 630)	Oxytetracycline	116/384	30%
	Penicillin and Gentamycin	94/384	24%
	Oxytetracycline and metronidazole	30/384	8.0%
	Metronidazole	27/384	7.0%
	Penicillin, gentamycin, and metronidazole	21/384	5.5%
Europe (n = 483)	Penicillin, gentamycin, and metronidazole	55/210	26%
	Penicillin and gentamicin	50/210	23%
	Metronidazole	45/210	21%
Australia (n = 141)	Penicillin, gentamicin, and metronidazole	29/73	40%
	Metronidazole	20/73	27%
	Penicillin and gentamicin	13/73	18%
Japan (n = 35)	Cephalothin	22/26	84%
	Cephalothin and metronidazole	3/26	11%
Latin America (n = 149	Penicillin and gentamicin	41/97	42%
	Gentamicin	26/97	27%
	Penicillin, gentamicin, and metronidazole	16/97	16%

Differences in the proportion of horses treated with antimicrobial drugs among the seasons were not observed in Europe, Japan, and Australia (P > 0.05). In North America, the use of antimicrobial drugs was higher during the summer (67%, 127/189) than in the fall (55%, 76/137) (P = 0.03). No other seasonal differences were observed in North America. This increase in antimicrobial drug use in the summer was associated with an increase in the use of oxytetracycline (data not shown).

#### Antimicrobial therapy, leukopenia, and SIRS

In total, 1199 horses had information regarding the total WBC and antimicrobial treatment. Of those, 70% (386/548) and 30% (162/548) of horses with leukopenia did and did not receive antimicrobial drugs, respectively. In addition, 51% (342/665) and 49% (323/665) of horses without leukopenia did and did not receive antimicrobial drugs, respectively. Horses with leukopenia had higher odds of being administered antimicrobial drugs (OR: 2.264, 95%CI: 1.754 to 2.921; P < 0.001) (**Table 5**). The treatment regimens administered to leukopenic and non-leukopenic horses are presented in **Table 6**.

**Table 5:** Multivariable mixed model evaluating the association between the presence of leukopenia, systemic inflammatory response syndrome (SIRS) and their combination and the administration of antimicrobial drugs in horses with acute diarrhea.

		95% CI				95% CI	
	Estimate	Lower	Upper	p-value	OR	Lower	Upper
Model 1							
*Random effects*							
Institution	0.600	0.288	1.250	0.008	-	-	-
*Fixed effects*							
Leukopenia							
No (n = 665)	Referent						
Yes (n = 548)	0.806	0.552	1.060	< 0.001	2.239	1.737	2.885
Model 2							
*Random effects*							
Institution	0.622	0.292	1.324	0.010	-	-	-
*Fixed effects*							
SIRS							
No (n = 319**)**	Referent						
Yes (n = 800)	0.933	0.652	1.214	<0.001	2.542	1.919	3.368
Model 3							
*Random effects*							
Institution	0.525	0.204	1.351	0.038	-	-	-
*Fixed effects*							
SIRS + leukopenia							
No, only SIRS (n = 400)	Referent						
Yes (n = 360)	0.378	0.053	0.704	< 0.001	1.460	1.054	2.022

Note: Age and sex were included as fixed effects in all 3 models, but the effect was not significant (p > 0.05)

**Table 6:** Antimicrobial treatment regimens administered to leukopenic and non-leukopenic horses with acute diarrhea presented to 26 institutions from North America, Latin America, Europe, Australia, and Japan.

Health condition	Antimicrobial regimen	n	%
*All geographic regions*			
Leukopenic n = 548	Penicillin and gentamicin	110/386	28%
	Penicillin, gentamicin, and metronidazole	74/386	19%
	Metronidazole	47/386	12%
	Oxytetracycline	42/386	11%
Non-leukopenic n = 665	Oxytetracycline	76/342	22%
	Penicillin and gentamicin	66/342	19%
	Penicillin, gentamicin, and metronidazole	46/342	13%
	Metronidazole	39/342	11%
*All geographic regions but North America*			
Leukopenic n = 287	Penicillin, gentamicin, and metronidazole	63/193	32%
	Penicillin and gentamicin	45/193	23%
	Metronidazole.	34/193	18%
Non-leukopenic n = 350	Penicillin, gentamicin, and metronidazole	36/173	22%
	Penicillin and gentamicin	39/173	20%
	Metronidazole	28/173	16%
	Cephalothin	14/173	8%

Leukopenia was defined as a total withe blood cell count < 5,300 cells/μL.

Information regarding SIRS criteria and antimicrobial treatment rates was available for 1119/1438 of the horses. Of those, 68% (542/800) and 45% (136/319) of the horses meeting and not meeting SIRS criteria were administered antimicrobial drugs, respectively. In addition, 32% (253/800) and 55% (175/319) of the horses meeting and not meeting SIRS criteria were not treated with antimicrobial drugs, respectively. Horses meeting SIRS criteria had higher odds of being treated with antimicrobial drugs (OR: 2.542, 95%CI: 1.919 to 3.368; P < 0.001). A total of 73% (262/360) of the horses meeting SIRS criteria and leukopenia were administered antimicrobial drugs, while 64% (257/400) of the horses meeting SIRS criteria but without leukopenia were administered antimicrobial drugs. Horses meeting SIRS criteria with leukopenia had higher odds of being treated with antimicrobial drugs than those meeting SIRS criteria without leukopenia (OR: 1.460, 95%CI: 1.054 to 2.022; P < 0.001) (**Table 5**). For all multivariable mixed models, the random effect of the institution was significant (P < 0.05), but the fixed effects of sex and age were not significant (P > 0.05).

An analysis excluding horses from North America was conducted because oxytetracycline was the most common drug used in non-leukopenic horses, likely due to the possibility of *N*. *risticii*. This analysis revealed that leukopenic horses had higher odds of being administered antimicrobial drugs than those without leukopenia (OR: 2.004, 95%CI: 1.410 to 2.849; P < 0.001) and horses meeting SIRS criteria had higher odds of being treated with antimicrobial drugs than those not meeting SIRS criteria (OR: 3.034, 95%CI: 2.027 to 4.544; P < 0.001). Horses meeting SIRS criteria with leukopenia had higher odds of being treated with antimicrobial drugs than those meeting SIRS criteria and leukopenia, but this association was not statistically significant (OR: 1.167, 95%CI: 0.739 to 1.843; P = 0.507). For all multivariable mixed models, the random effect of the institution was significant (P < 0.05), but the fixed effect of sex and age was not (P > 0.05).

#### Probiotics during hospitalization

Probiotics were administered to 15% (215/1418; 95%CI: 13% to 17%) of horses. The most commonly administered probiotics were products containing lactic acid-producing bacteria (LAB) (67%, 143/215) or *Saccharomyces* spp. (18%, 38/215). Probiotics containing LAB were administered to 100% of the horses treated in Japan (**Table 3**).

#### Polymyxin B and pentoxifylline during hospitalization

Administration of polymyxin B was reported in 13% (187/1438; 95%CI: 11% to 15%) of the horses. The proportion of horses administered polymyxin B during hospitalization was higher in Japan than in the other geographic areas (**Table 1**). The proportion of horses that met the criteria for SIRS treated with polymyxin B (16%, 134/794) was higher than that of horses not meeting the SIRS criteria (10%, 32/316) (P < 0.01). In total, 17% (95/449) and 13% (75/600) of the leukopenic and non-leukopenic horses were treated with polymyxin B (P = 0.02). A total of 19% (87/464) of the horses meeting SIRS criteria and leukopenia were administered polymyxin B, while 15% (44/284) of the horses meeting SIRS criteria but without leukopenia were administered this drug (P = 0.26).

Administration of pentoxifylline during hospitalization was reported in 8% (116/1438; 95%CI: 6.9% to 9.9%) of horses. The proportion of horses that met the criteria for SIRS treated with pentoxifylline (10%, 83/792) was higher than that of horses not meeting the SIRS criteria (3%, 10/316) (P < 0.001). In total, 8% (42/544) and 8.3% (50/598) of the leukopenic and non-leukopenic horses were treated with pentoxifylline (P = 0.78), respectively. A total of 8% (39/464) of the horses meeting SIRS criteria and leukopenia were administered pentoxifylline, while 13% (38/282) of the horses meeting SIRS criteria but without leukopenia were administered this drug (P = 0.03).

#### Gastroprotectants

Information regarding administering gastroprotectants at any time during hospitalization was available for 1419 horses, with 44% (626/1419; 95%CI: 40% to 46%) being administered a type of gastroprotectant or a combination. The proportion of horses administered gastroprotectants varied from 0% (Japan) to 78% (85/108, Copenhagen) (**Table 3**). The proportion of horses treated with gastroprotectants was higher in Latin America than in the other geographic areas (**Table 1**).

#### Digital cryotherapy

Information regarding digital cryotherapy was available for 1419 horses. Digital cryotherapy was used in 22 institutions, with 34% (489/1435; 95%CI: 32% to 37%) of the horses having digital cryotherapy. The proportion of horses treated with digital cryotherapy was higher in Australia than in other geographic areas (**Table 1**).

The techniques for digital cryotherapy were documented in 353 cases. Digital cryotherapy techniques included fluid bags filled with ice (176/353), ice boots (117/353), ice packs on the foot only or distal limb only or distal limb and foot (31/353), ice bandages (28/353) and coronet sleeve (1/353). In Australia, the most common technique for digital cryotherapy was fluid bags (52%, 53/101), followed by ice boots (44%, 44/101) and ice packs (4%, 4/101), while in Europe, the most common technique was ice bandages (43%, 28/64), followed by fluid bags (22%, 14/64), ice packs (20%, 13/64) and ice boots (15%, 9/64). In North America, fluid bags were the most common technique used for digital cryotherapy, with 66% (109/166) being managed with this technique, followed by ice boots (33%, 56/166). In Latin America, ice packs were used in 63% (14/22) of the horses, ice boots in 32% (7/22), and coronet sleeves in 5% (1/22).

#### Toxin binding agents and antidiarrheal drugs

In total, 36% (515/1438; 95%CI: 34% to 39%) of horses were treated with a toxin-binding agent, including di tri octahedral smectite (94%, 486/515), charcoal (1%, 8/515), montmorillonite clay (6/515, 1%), bismuth subsalicylate (1%, 6/515), a combination of montmorillonite clay and charcoal (1%, 7/515) and kaolin-pectin (0.3%, 2/515) (**Table 3**). The proportion of horses treated with smectite varied from 2% (UG) to 91% (Adelaide).

## Discussion

This retrospective international multicenter study described treatment approaches used in 1438 diarrheic horses in 26 institutions worldwide. We showed that antimicrobial drugs are administered to 55% of diarrheic horses within the first 24 hours of admission. A combination of penicillin and gentamicin was the most used antimicrobial therapy administered. Horses with leukopenia or meeting systemic inflammatory response syndrome criteria had higher odds of being administered antimicrobial drugs. However, approximately 50% of the horses without leukopenia received antimicrobial drugs during the first 24 hours of admission. In addition to fluids, other commonly administered treatments (in descending order) included gastroprotectants, digital cryotherapy, toxin-binding agents, antidiarrheic drugs, probiotics, polymyxin B, plasma transfusion, and pentoxifylline. This study revealed differences in the treatment approaches for diarrheic horses between hospitals, highlighting the lack of standardized guidelines and evidence-based recommendations for managing these cases.

### Fluid therapy on admission

#### Hypertonic saline

HSS was used in 15% of horses, with increasing use associated with a higher perceived level of dehydration. Experimentally, administering HSS to euvolemic horses improves systolic cardiac function for 40 to 60 minutes and positively affects blood pressure for 30 minutes [13, 14]. In clinical settings, HSS is superior to both isotonic saline and synthetic colloids in the correction of haemoconcentration; however, in horses undergoing exploratory celiotomy for colic, a survival benefit is not observed [15]. In contrast, synthetic colloids result in a higher cardiac index in horses with colic compared to HSS [16]. In addition, administration of HSS in combination with synthetic colloids fails to ameliorate the effects of experimentally induced endotoxemia in anesthetized horses on systemic vascular resistance (SVR), mean arterial pressure, blood L-lactate concentrations, or arterial oxygenation [16]. Therefore, the clinical benefits of administering HSS to horses with acute diarrhea and dehydration remain to be proven.

#### Bolus of isotonic crystalloids

Crystalloid fluid boluses were generally given to more dehydrated horses, although the rate of administration to horses not considered dehydrated was 38%. It is unknown if this high rate of bolus therapy in reported euvolemic horses was due to clinical suspicion of impending fluid losses associated with diarrhea or a tendency towards overhydration and its tolerance by horse and clinician alike. Despite this, however, proxies of dehydration and hypovolemia were associated with higher odds of fluid bolus administered at admission. There is considerable evidence in humans that fluid overload is associated with adverse outcomes, including increased mortality [17, 18]; however, information regarding fluid overload and outcomes in horses is scarce and limited by the retrospective experimental design or lack of statistical power of the studies [19, 20].

The beneficial effects of bolus fluid therapy in human medicine have recently been questioned. In children with shock, boluses are associated with a higher mortality rate [21]. In contrast, 1L of saline over 30 minutes in healthy humans led to a drop in cardiac output compared to the same volume over 120 minutes [22]. The evidence for the beneficial cardiovascular effects of fluid boluses in horses is even lower. Healthy anesthetized horses had better femoral arterial flow when they received a 20 mL/kg bolus of Hartmann’s solution in addition to dobutamine, over dobutamine alone [23]. The type of fluid bolus administered likely reflects the commercial availability of larger fluid bags rather than clinical indication in most cases. Despite previous evidence suggesting a benefit to balanced electrolyte solutions over normal saline for resuscitation of adults and children on mortality rates and acute renal injury, the actual effect is small or possibly zero [24]. Still, its importance in resuscitation in horses with diarrhea is unknown.

#### Synthetic colloids

Ten percent of horses received synthetic colloids at admission, with increasing rates in horses perceived to be more dehydrated or with lower TS/TPP concentrations. Synthetic colloids are effective at increasing colloid oncotic pressure (COP) in horses [25, 26] and are superior to crystalloid fluids for the maintenance of COP, although this effect is dose-dependent [27]. Evidence shows that synthetic colloids are superior to hypertonic saline in improving cardiac index in horses with colic [16]. However, combining HSS and synthetic colloids does not ameliorate the cardiovascular effects of experimental endotoxemia in horses [28]. This study did not determine the use of a combination of these products. The survival proportion of horses with colitis treated with synthetic colloids is lower (47%, 11/23) than horses administered fresh frozen plasma (80%, 54/69) despite similar disease severity at admission, suggesting that the use of natural colloids could be superior to treatment with synthetic colloids in horses with colitis [29]. Other concerns regarding the use of colloids in critically ill humans, namely coagulopathies and acute kidney injury, do not appear to be major concerns in horses, at least in the populations studied thus far [30–33]. The clinical benefits of colloid administration to critically ill horses, especially those with diarrhea, are yet to be studied.

#### Plasma transfusion

Plasma was used in this study in a small percentage (13%) of horses, with a generally small median volume (4 litres). Although limitations of this retrospective study prevented us from being able to accurately report the dose of plasma in a mL/kg, the breed prevalence and the median volume administered indicated that the administered volumes of plasma were unlikely to make a substantial difference in COP of these horses [34]. The use of plasma in horses with hypoproteinemia and low COP also remains controversial. Potential benefits associated with the use of plasma versus synthetic colloids include the provision of albumin, which in addition to improving COP, also serves as a transporter for both exogenous (i.e., drugs) and endogenous (i.e., hormones) compounds [35], plays an essential role in maintaining the glycocalyx integrity [36], and has antioxidant and anti-inflammatory properties, such as modulating inflammation through the binding of LPS [37, 38]. Additionally, theoretically, plasma can also provide the patient with additional components, including clotting factors and antithrombin. However, disadvantages of using plasma include the risk of a transfusion reaction, worsening of the interstitial edema, transmission of blood-borne diseases (e.g., equine hepacivirus), cost, and the inconvenience of frozen storage. There are two small-scale retrospective studies evaluating the outcome of horses with colitis receiving plasma, with one study noting that horses that received plasma were less likely to survive hospitalization [34] and another finding that horses that received plasma were more likely to survive than those receiving synthetic colloids [29]. This study did not determine the association between the use of plasma and survival, which can be investigated by a large-scale prospective multicenter study.

#### Antimicrobial therapy

The use of antimicrobial drugs in horses with diarrhea remains controversial, and guidelines for their usage are lacking. Many causes of diarrhea in horses, except *N*. *risticii*, are not due to defined bacterial pathogens that require antimicrobial therapy [39]. This, combined with the potential adverse effects of antimicrobials on an already disrupted microbiota and the increased risk of systemic adverse effects (i.e., nephrotoxicity) with some antimicrobials in the face of hemodynamic compromise [40, 41], led many clinicians to refrain from using antimicrobial drugs in horses with diarrhea. In contrast, other clinicians advocate their use in horses with evidence of bacterial translocation and systemic compromise (i.e., those horses meeting SIRS criteria and/or those with leukopenia) [42]. This study found that horses with leukopenia and meeting SIRS criteria were more likely to receive antimicrobials than those without, however many horses that were not leukopenic and did not meet SIRS criteria also received broad-spectrum antimicrobial therapy. The retrospective nature of this study did not allow for capturing the clinicians’ rationale for using antimicrobial drugs in these horses nor whether the use of antimicrobials influenced survival. However, the overall high prevalence of antimicrobial use in horses with diarrhea, even without evidence of systemic compromise, warrants further consideration. Interestingly, in Europe, where the use of some antimicrobial drugs is more heavily regulated, there was an overall lower prevalence of use compared to countries where their use is less tightly monitored and controlled. Despite the lower prevalence of antimicrobial use in Europe, there was no difference in survival between continents. Of the antimicrobials used, the most common combinations were broad spectrum (i.e., penicillin/gentamicin or penicillin/gentamicin/metronidazole), except in North America, where the most common antimicrobial regimen reported was oxytetracycline. This is likely due to the prevalence of *N*. *risticii*, a known bacterial pathogen that causes colitis and requires treatment with tetracyclines [39, 43].

#### Probiotics

Probiotics were administered to 15% (215/1418) of horses. Probiotics, defined as live microorganisms that provide a health benefit to the host when delivered in an adequate amount [44], are often perceived as a benign or helpful treatment, but their use has raised controversy in recent years. Much of the controversy surrounding commercial probiotics is associated with significant discrepancies between the label claim and actual contents [45–47]. Other concerns with commercial probiotics include the potential for administering bacteria containing transferrable antimicrobial resistance genes [45, 48], the association with the development of diarrhea (rather than prevention of) in foals [49–51], and questions regarding the bacteria that should be administered to horses with colitis [52]. Although no studies report adverse effects of probiotics in adult horses with diarrhea, the evidence of the positive effects associated with their use in adult horses is limited to questionable [53], likely due partly to persistent issues with quality control [45–47]. While probiotics can potentially improve intestinal health, more research is needed to maximize their potential for use in horses with diarrhea.

#### Polymyxin B and pentoxifylline

Polymyxin B and pentoxifylline were administered to 13% and 8% of horses with diarrhea, respectively. Polymyxin B is used as an anti-endotoxic medication with demonstrated anti-inflammatory and anti-endotoxic effects in horses [54, 55]. There is emerging evidence in human medicine that polymyxin B hemoperfusion can improve survival in sepsis [56]; although, more recent trials that have questioned the benefit in septic patients due to issues with methodology [57, 58]. Pentoxifylline has less objective evidence supporting its use in septic or endotoxic conditions in horses. In horses with experimental endotoxemia, pentoxifylline lowers thromboxane B_2_ concentrations [59], respiratory rate and rectal temperature [60], and, when combined with flunixin meglumine, lower WBCs and higher interleukin-6 concentrations [61]. However, it should be noted that polymyxin B is associated with an increased concentration of creatinine and transient ataxia in healthy horses [41]. As yet, no evidence suggests a survival benefit for either drug in horses with diarrhea.

#### Gastroprotectants

In total, 44% of horses received gastroprotectants in this study. Omeprazole was the most used gastroprotectant in the participating institutions. However, preliminary data shows that only 20% of horses hospitalized with colitis have gastric disease on *post-mortem* examination [62], suggesting that a critical assessment of the administration of gastroprotectants in horses with diarrhea is required. In addition, in human medicine, evidence is conflicting on whether omeprazole and other proton pump inhibitors increase the risk of *C*. *difficile*-associated disease [63]. In foals, acid-suppression therapy is also associated with an increased risk of diarrhea [64], although this effect has not been replicated in adults.

#### Cryotherapy

Approximately one-third of horses received digital cryotherapy for the prevention of laminitis. Cryotherapy reduces the incidence and grade of laminitis in horses with colitis across various populations, and aetiologic agents studied [5]. Experimental evidence suggests that digital cryotherapy using fluid bags filled with ice is the most effective method of application [65]. In approximately 50% of the cases, this method of digital cryotherapy was applied, with ice boots being the second most used technique. The timing of application was unknown in this study, however recent evidence suggests that cryotherapy can have a beneficial effect even after the onset of laminitis [66].

#### Toxin-biding agents

The most used toxin-binding agent in this study was di-tri-octahedral (DTO) smectite. In vitro, DTO smectite binds endotoxin, *C*. *difficile* toxins A and B, and *Clostridium perfringens* enterotoxin without affecting clostridial organism growth [67]. In a clinical setting, a single non-blinded randomized clinical trial showed that the administration of DTO smectite orally every 24h for 3 days beginning 4 h post-operatively decreased the prevalence of postoperative diarrhea from 41% (controls, n = 30) to 11% (cases, n = 37). Still, DTO smectite did not alter any clinical variables, total WBC, or survival of the horses [68]. Although this study showed a significant benefit on post-operative diarrhea rates, the results of the study were limited by the small number of animals enrolled, the use of a complex and subjective score for classification of post-operative diarrhea, and the inclusion of horses with different surgical lesions at different risks of developing post-operative diarrhea. Thus, the potential beneficial effects of DTO smectite in horses with acute diarrhea are yet to be determined.

#### Limitations

Our study has several limitations, most notably its retrospective design, non-standardized data collection, and lack of clinical signs and treatments categorization. This prevented the assessment of the effect of individual treatment variations on survival. Thus, the positive or negative impact of these treatments cannot be inferred. Second, due to the retrospective nature of this study, the clinician rationale for the treatments assessed in this study could not be determined. Therefore, multicentered prospective randomized clinical trials are needed to determine the beneficial effect of the different therapies used in horses with diarrhea. In addition, qualitative research assessing clinicians’ perspectives and attitudes regarding the different therapeutic regimens can provide some insights into the clinical decision-making by veterinarians when treating horses with diarrhea. In addition, accumulating quantitative and qualitative data can lead to the development of evidence-based guidelines for managing diarrheic horses. Finally, the study gathered data from diarrheic horses presented to referral institutions, potentially biasing the results towards more severely affected animals. Thus, these findings should be applied cautiously to different populations of horses. The number of cases varied significantly between institutions, likely reflecting the population each institution serves. Despite this, the study provided a comprehensive analysis of the treatment approach used in diarrheic horses worldwide.

In conclusion, this study found that a wide variety of treatments are employed in horses with diarrhea. Unfortunately, many are used without solid evidence in the literature for their use in horses with acute diarrhea. Further work is needed to determine what treatments are effective or even potentially harmful in horses which would be best accomplished by multicenter prospective clinical trials.
